# Subgenotyping of Genotype C Hepatitis B Virus: Correcting Misclassifications and Identifying a Novel Subgenotype

**DOI:** 10.1371/journal.pone.0047271

**Published:** 2012-10-15

**Authors:** Weifeng Shi, Chaodong Zhu, Weimin Zheng, Wei Zheng, Cheng Ling, Michael J. Carr, Desmond G. Higgins, Zhong Zhang

**Affiliations:** 1 Guangzhou Institute of Advanced Technology, Chinese Academy of Sciences, Guangzhou, China; 2 Key Laboratory of Zoological Systematics and Evolution, Institute of Zoology, Chinese Academy of Sciences, Beijing, China; 3 Shenzhen Institute of Advanced Technology, Chinese Academy of Sciences, Shenzhen, China; 4 Graduate University of Chinese Academy of Sciences, Beijing, China; 5 National Virus Reference Laboratory, University College Dublin, Dublin, Ireland; 6 The Conway Institute of Biomolecular and Biomedical Research, University College Dublin, Dublin, Ireland; 7 Department of Basic Medicine, Taishan Medical College, Taian, Shandong, China; University of Kansas Medical Center, United States of America

## Abstract

**Background:**

More than ten subgenotypes of genotype C Hepatitis B virus (HBV) have been reported, including C1 to C16 and two C/D recombinant subgenotypes (CD1 and CD2), however, inconsistent designations of these subgenotypes still exist.

**Methodology/Principal Findings:**

We performed a phylogenetic analysis of all full-length genotype C HBV genome sequences to correct the misclassifications of HBV subgenotypes and to study the influence of recombination on HBV subgenotyping. Our results showed that although inclusion of the recombinant sequences changed the topology of the phylogenetic tree, it did not affect the subgenotyping of the non-recombinant sequences, except subgenotype C2. In addition, most of the subgenotypes have been properly designated. However, several misclassifications of HBV subgenotypes have been identified and corrected. For example, C11 proposed by Utsumi and colleagues in 2011 was found to be grouped with C12 proposed by Mulyanto and colleagues. Two sequences, GQ358157 and GU721029, previously designated as C6 have been re-designated as C12 and C7, respectively. Moreover, a quasi-subgenotype C2 was proposed, which included the old C2, several previously unclassified sequences and previously designated C14. In particular, we identified a novel subgenotype, tentative C14, which was well supported by phylogenetic analysis and sequence divergence of >4%.

**Conclusions/Significance:**

A number of misclassifications in the subgenotyping of genotype C HBV have been identified in this study. After correcting the misclassifications, we proposed a better classification for the subgenotyping of genotype C HBV, in which a novel quasi-subgenotype C2 and a novel subgenotype, tentative C14, were described. Based on this large-scale analysis, we propose that a novel subgenotype should only be reported after a complete comparison of all relevant sequences rather than a few representative sequences only.

## Introduction

Ten genotypes (from genotype A to J) [1]–[3] and more than 30 subgenotypes [4] of HBV have been identified based on the general rule that different genotypes should diverge by at least 8% [5] and different subgenotypes should diverge by at least 4% over the entire genome [6]. Other rules for HBV genotyping and subgenotyping include the monophyletic nature of the genotypes and subgenotypes on a phylogenetic tree and high bootstrap support [7], [8].

To date, genotype C has the largest number of reported subgenotypes, with at least 16 subgenotypes identified. In early 2004, Huy et al. found that genotype C could be classified at least two subgenotypes C1 and C2 [9]. Also in 2004, Norder et al. divided genotype C into four subgenotypes: C1 from East Asia, C2 mostly from China and Southeast Asia, C3 from Oceania and C4 from aborigines from Australia [10], [11]. Subgenotype C5 was isolated from patients from the Philippines in 2006 [12]. Subgenotype C6 was first proposed by analyzing the S gene sequences and preC-C gene sequences from Papua, Indonesia [13], which was later confirmed by complete genome sequences in 2009 [14]. Almost at the same time, a virus strain isolated from the Philippines was also defined as subgenotype C6 [15]. After a comparison between these two C6 subgenotypes, the one from the Philippines was renamed as C7 [7], [16]. However, some viruses from Nusa Tenggara, Indonesia were also named as subgenotype C7 by Mulyanto and colleagues [17]. To avoid potential confusion in the delimitation of subgenotypes, Mulyanto and colleagues renamed their C7 as C8 in 2010 [18]. In addition, they also proposed a novel subgenotype C9, which they originally reported as an unclassifiable subgenotype [18]. Subgenotype C10 was also isolated from Indonesia where a few novel subgenotypes, such as B7, B8 and C7 to C9, were identified [18]. In 2011, two independent research groups named some viruses isolated from Indonesia as C11, respectively [19], [20]. Moreover, Mulyanto et al. reported another novel subgenotype C12, which has the same geographical origin as C11s and many other HBV subgenotypes [19]. Recently, Mulyanto et al. further described four novel subgenotypes C13 to C16 [21]. These four subgenotypes were also isolated from Papua, Indonesia. Finally, two more subgenotypes associated with C/D recombination, CD1 and CD2, were isolated from Tibet, China [22]–[24].

Different genotypes usually have distinct geographical distributions [3]. However, both genotypes B and C are prevalent in Asia and Oceania [3]. This has led to potential recombination between B and C due to co-infection or super-infection [25], [26]. Several genotype C viruses have been reported to be recombinants. For example, a C13 strain from Indonesia was identified to be a C13/B3 recombinant [21]. Also in Indonesia, a strain of subgenotype C12 was proved to be a C/G recombinant [21]. In addition, some C/D recombinants have also been isolated from China [22], [23].

A number of problems in the subgenotyping of genotype C HBV have been reported [7]. First, there was reported incongruence in C1 and C2 proposed respectively by Huy et al. and Norder et al. [27]. Although Schaefer and colleagues suggested that the designation proposed by Huy et al. should be used [7], subgenotype C2 proposed by Huy et al. was not a monophyly [9]. Second, there were two C6 subgenotypes proposed by different research groups [13], [15], though the one from the Philippines was subsequently renamed as C7 [16]. Third, as mentioned above, two new subgenotypes were named as C11 respectively in 2011 [19], [20]. Fourth, including recombinant sequences into phylogenetic analysis sometimes might change the topology of the tree and increase the sequence divergences estimated. In addition, including recombinant sequences may also change (mostly increase) the sequence divergence. Therefore, recombination played a potential role in HBV subgenotyping. However, unfortunately, most previous studies failed to take recombination into consideration when they designated novel subgenotypes.

In order to determine how the recombination influences HBV subgenotyping, to correct the known and potential unidentified misclassifications in the subgenotyping of genotype C HBV, and to establish a better classification, we analyzed a large number of full-length genotype C HBV sequences using a phylogenetic approach.

## Materials and Methods

In our previous report, 1214 sequences have been identified to be of genotype C, including 96 potential recombinants [28]. All these sequences were selected to compose a new dataset for further analysis. A second dataset excluding the recombinant sequences was also composed. In addition, a sequence of genotype B (GenBank accession number: D00329) was included in the two datasets and used as an outgroup. Information of these sequences, such as subgenotype and recombination, was extracted from GenBank annotations. An extensive literature review for sequences with references available in Pubmed was carried out to obtain their subgenotype and recombination information, which was then used in defining the subgenotypes.

Phylogenetic analysis of the two datasets was carried out using RAxML [29] under the GTRCAT approximation [30] and random starting trees. One thousand rapid bootstrap replicates were performed with all other parameters set to default. Trees were visualized and analyzed using Dendroscope [31]. The trees are available as Figures S1 and S2.

The mean nucleotide divergence (mean ± SD) between different subgenotypes was calculated using Mega 5 [32] with the Kimura 2-parameter model [33]. In order to obtain consistent and reliable sequence divergence values, 500 bootstrap replicates were applied.

## Results

**Figure 1:**
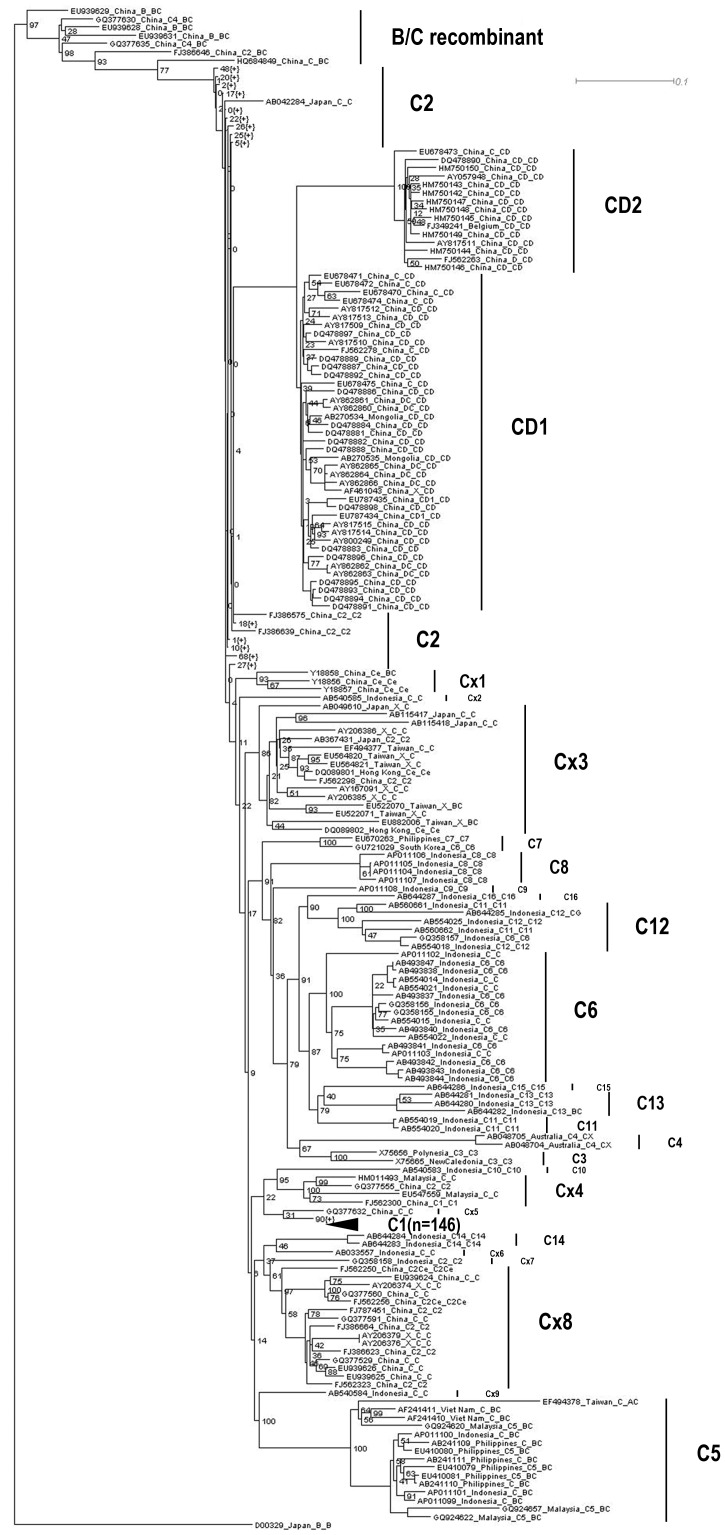
A simplified phylogenetic tree of all genotype C HBV sequences.

Phylogenetic analysis of all genotype C sequences showed that four subgenotypes, CD1, CD2, C4, and C5, were inter-genotype recombinants (Figure 1) [22]–[24], [28]. CD1 and CD2 have been proposed as recombinant subgenotypes of genotype C [23]. They were composed of C/D recombinants. Sequence divergence between CD1 and CD2 and that between CD2 and C2 were 4.1% and 5.7%, respectively (Table 1). However, sequence divergence between CD1 and C2 was 3.8% (Table 1), less than 4% (the general rule to define a new subgenotype). C4 was associated with inter-genotype recombination between genotype C and an unknown genotype. Sequences of C5 were mostly B/C recombinants, with one A/C recombinant.

**Table 1:** Mean percentage of nucleotide divergences over the entire genome of HBV between different subgenotypes/suspect lineages calculated using all genotype C sequences.

	CD2	CD1	C2	C×1	C×2	C1	C×3	C10	C×4	C×5	C×6	C×7	C7	C4	C3	C11	C6	C12	C9	C8	C5
CD2		0.3	0.4	0.5	0.5	0.5	0.4	0.6	0.4	0.5	0.4	0.4	0.5	0.5	0.5	0.5	0.5	0.5	0.5	0.5	0.5
CD1	4.1		0.3	0.4	0.4	0.4	0.3	0.5	0.4	0.4	0.3	0.3	0.4	0.5	0.4	0.5	0.4	0.4	0.4	0.4	0.4
C2	5.7	3.8		0.2	0.3	0.3	0.2	0.4	0.3	0.3	0.2	0.2	0.3	0.4	0.3	0.4	0.3	0.3	0.3	0.3	0.3
C×1	6.2	4.4	3.3		0.3	0.4	0.3	0.5	0.3	0.4	0.3	0.3	0.3	0.5	0.4	0.4	0.4	0.3	0.3	0.4	0.4
C×2	6.1	4.6	3.5	4.0		0.4	0.3	0.4	0.4	0.4	0.3	0.3	0.3	0.5	0.4	0.5	0.4	0.4	0.4	0.4	0.4
C1	6.8	5.3	4.6	5.1	4.9		0.3	0.4	0.3	0.3	0.3	0.3	0.4	0.5	0.4	0.4	0.4	0.4	0.4	0.4	0.4
C×3	5.1	3.8	2.8	3.4	3.5	3.1		0.4	0.3	0.4	0.3	0.3	0.3	0.5	0.4	0.4	0.4	0.4	0.3	0.4	0.4
C10	7.6	6.4	5.4	5.9	5.2	5.5	5.1		0.4	0.5	0.4	0.4	0.5	0.6	0.5	0.6	0.5	0.5	0.5	0.5	0.5
C×4	6.5	5.2	4.4	4.9	4.7	4.8	3.9	5.2		0.3	0.3	0.3	0.3	0.5	0.3	0.4	0.4	0.3	0.3	0.4	0.4
C×5	6.3	4.4	3.5	3.9	4.0	4.5	3.5	5.1	4.3		0.3	0.3	0.4	0.5	0.4	0.5	0.4	0.4	0.4	0.4	0.4
C×6	6.2	4.8	3.8	4.4	4.2	4.7	3.6	5.6	4.7	3.9		0.2	0.3	0.5	0.3	0.4	0.3	0.3	0.3	0.4	0.4
C×7	6.4	4.5	3.7	4.2	4.0	4.9	3.7	5.6	4.7	4.1	4.3		0.3	0.4	0.3	0.4	0.3	0.3	0.3	0.4	0.3
C7	6.5	4.9	4.1	4.4	4.5	5.2	3.9	5.9	5.2	4.4	4.5	4.6		0.5	0.4	0.4	0.4	0.4	0.4	0.4	0.4
C4	7.6	7.0	7.0	7.3	7.2	8.0	6.7	8.4	7.5	7.4	7.2	7.2	7.3		0.4	0.5	0.5	0.4	0.5	0.5	0.5
C3	6.5	5.8	4.8	5.0	5.1	5.8	4.6	6.8	5.7	4.8	5.2	5.3	5.1	6.6		0.4	0.3	0.3	0.4	0.4	0.4
C11	7.0	6.0	5.1	5.4	5.4	6.1	4.8	6.9	6.0	5.5	5.4	5.8	5.2	6.8	4.8		0.3	0.4	0.4	0.4	0.5
C6	7.3	6.0	5.0	5.3	5.2	6.2	5.0	6.8	6.0	5.3	5.2	5.6	5.3	7.1	4.7	4.2		0.3	0.3	0.4	0.4
C12	7.3	6.1	5.2	5.5	5.7	6.4	5.1	7.1	6.0	5.3	5.3	5.7	5.2	7.1	5.1	4.8	4.9		0.3	0.4	0.4
C9	6.5	5.2	4.3	4.5	4.5	5.5	4.2	6.0	5.1	4.3	4.9	4.8	4.2	6.7	4.6	4.8	4.8	4.9		0.4	0.4
C8	6.9	5.5	4.6	5.0	4.8	5.7	4.5	6.6	5.8	4.9	4.9	5.1	4.6	6.9	5.0	5.3	5.2	5.3	4.6		0.4
C5	7.8	6.6	5.9	6.1	6.4	6.7	5.7	7.3	6.7	5.8	6.2	6.2	6.3	8.8	6.5	7.2	7.1	7.2	6.7	6.3	

**Figure 2:**
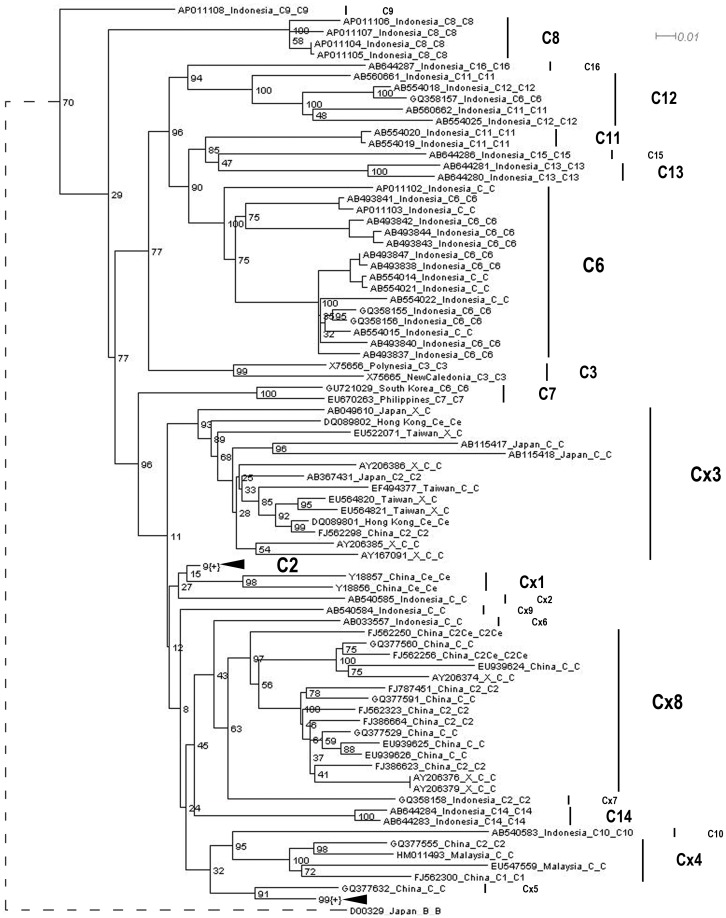
A simplified phylogenetic tree of all non-recombinant genotype C HBV sequences.

It should be noted that, although inclusion of the recombinants did not influence HBV subgenotyping greatly (in other words, the clustering of non-recombinant subgenotypes was not changed greatly), it did change the topology of the phylogenetic tree (Figures 1 and 2). For example, C2 was closer to the root of the tree than C9 in the phylogenetic tree built using all the genotype C sequences (Figure 1). However, in the tree estimated using non-recombinant sequences only, C9 was the closest subgenotype to the root of tree (Figure 2).

Our results revealed that most of the subgenotypes were properly designated. Subgenotypes C1, C3, C6, C7, C8, C9, C10, C11 (proposed by Mulyanto et al. [19]), C12, C13, C15 and C16 were monophyletic (Figures 1 and 2). Sequence divergence between any two of the above subgenotypes was greater than 4% (Tables 1 and 2). Therefore, these subgenotypes were properly designated and should be maintained.

**Table 2:** Mean percentage of nucleotide divergences over the entire genome of HBV between different subgenotypes/suspect lineages calculated using non-recombinant genotype C sequences.

	C9	C3	C12	C11	C6	C8	C×7	C×5	C×2	C2	C×1	C×6	C1	C×3	C10	C×4	C7
C9		0.4	0.3	0.4	0.3	0.4	0.3	0.4	0.4	0.3	0.4	0.3	0.4	0.3	0.5	0.3	0.4
C3	4.6		0.3	0.4	0.3	0.4	0.3	0.4	0.4	0.3	0.4	0.3	0.4	0.4	0.5	0.3	0.4
C12	4.9	5.1		0.4	0.3	0.4	0.3	0.4	0.4	0.3	0.4	0.3	0.4	0.4	0.5	0.3	0.4
C11	4.8	4.8	4.8		0.3	0.4	0.4	0.5	0.5	0.4	0.5	0.4	0.4	0.4	0.6	0.4	0.4
C6	4.8	4.7	4.9	4.2		0.4	0.4	0.4	0.4	0.3	0.4	0.3	0.4	0.4	0.5	0.4	0.4
C8	4.6	5.0	5.3	5.3	5.2		0.4	0.4	0.4	0.3	0.4	0.4	0.4	0.4	0.5	0.4	0.4
C×7	4.7	5.2	5.6	5.7	5.5	5.0		0.3	0.3	0.2	0.3	0.2	0.3	0.3	0.4	0.3	0.3
C×5	4.3	4.8	5.3	5.5	5.3	4.9	4.0		0.4	0.3	0.4	0.3	0.3	0.4	0.5	0.3	0.4
C×2	4.5	5.1	5.7	5.4	5.2	4.8	3.9	4.0		0.3	0.3	0.3	0.4	0.3	0.4	0.4	0.3
C2	4.3	4.8	5.2	5.1	5.0	4.6	3.6	3.5	3.5		0.2	0.2	0.3	0.2	0.4	0.3	0.3
C×1	4.4	4.9	5.4	5.4	5.2	4.8	4.1	3.9	3.9	3.3		0.3	0.4	0.3	0.5	0.3	0.3
C×6	4.9	5.2	5.3	5.4	5.2	4.9	4.2	3.9	4.2	3.8	4.4		0.3	0.3	0.4	0.3	0.3
C1	5.4	5.8	6.4	6.1	6.2	5.7	4.8	4.5	4.9	4.5	5.0	4.7		0.3	0.4	0.3	0.4
C×3	4.2	4.6	5.1	4.8	5.0	4.5	3.6	3.5	3.5	2.8	3.4	3.6	3.1		0.4	0.3	0.3
C10	6.0	6.8	7.1	6.9	6.8	6.6	5.5	5.1	5.2	5.4	5.9	5.6	5.5	5.1		0.4	0.5
C×4	5.1	5.7	6.0	6.0	6.0	5.8	4.6	4.3	4.7	4.4	4.8	4.7	4.8	3.9	5.2		0.3
C7	4.2	5.1	5.2	5.2	5.3	4.6	4.5	4.4	4.5	4.1	4.5	4.5	5.1	3.9	5.9	5.2	

However, there were a few misclassifications. First, at the top part of the tree constructed using all genotype C sequences, three sequences (EU939628, EU939629 and EU939631) were previously defined as genotype B (Figure 1). We have demonstrated that they were B/C recombinants but closer to genotype C, and have corrected this information in a previous report [28]. Also at the top of the tree, there were three sequences from China, GQ377630, GQ377635 and FJ386646. Information extracted from GenBank showed the first two sequences belonged to subgenotype C4, and the third belonged to subgenotype C2 (Figure 1). Obviously, this information was not correct, because these sequences did not really cluster with C4 and C2 respectively; in fact, they have been already identified as B/C recombinants in our previous analysis [28].

Second, C11 has been named twice by two research groups respectively [19], [20]. Both of the trees revealed that C11 proposed by Utsumi and colleagues were actually clustered with C12 proposed by Mulyanto et al. [19], [20], supported by high bootstrap value (100%, Figures 1 and 2). Therefore, C11 proposed by Utsumi and colleagues should be renamed as C12.

Third, sequences of C6 fell into three parts in the two trees respectively (Figures 1 and 2). The first part was composed of 16 sequences isolated from Indonesia and has been labeled as subgenotype C6. However, in the second part, one sequence, GQ358157, previously defined as subgenotype C6 [34], fell into a cluster of subgenotype C12. In the third part, one sequence from South Korea, GU721029, was clustered with a C7 sequence from the Philippines [16]. Sequence divergences between the first C6 and other two parts, C12 and C7, were 5.1% and 5.3% respectively (Table 1). Therefore, the subgenotypes of sequences in the second and third parts were not properly defined. Instead, the subgenotype of GQ358157 should be C12, while that of GU721029 should be C7.

Fourth, both of the trees revealed that subgenotype C2 was not a monophyly and sequences previously designated as subgenotype C2 scattered into several parts in the trees (Figures 1 and 2). In addition, there was no subgenotype information for some sequences. To determine whether subgenotype C2 was properly defined and to classify the sequences without subgenotype information, we named a few suspect sequences or branches as C×1 to C×9 tentatively (Figures 1 and 2). However, sequence divergences between C2 and the tentative designations, C×1, C×2, C×3, C×5, C×6, C×8 and C×9 were less than 4% (Tables 1 and 2). By comparing the topologies of the two trees and mostly based on the phylogeny constructed using non-recombinant sequences (Figures 1 and 2), we proposed that subgenotypes C2, C×3, C×1, C×2, C×9, C×6, C×8, C×7 and C14 [21] composed a quasi-subgenotype C2 of Asian origin. Although sequence divergences between C×5 and several subgenotypes were less than 4% and that between C×5 and C2 was the lowest (2.8%), C×5 formed a monophyly with subgenotype C1 (Figures 1 and 2). In particular, it was supported with high bootstrap value of 91% (Figure 2). Therefore, C×5 should be classified as C1. Apart from C×5, sequence divergences between C×4 and other subgenotypes were always greater than 4% (Tables 1 and 2). Because lineage C×4 was a monophyly with high bootstrap value of 100%, it should be classified as a novel subgenotype. As previously defined C14 [21] has been classified into the quasi-subgenotype C2, we proposed that it should be named as the new C14 for continuous numbering. Then we calculated sequence divergences between non-recombinant subgenotypes in the novel classification (Table 3). Sequence divergences between the quasi-subgenotype C2, C1, new C14 and any of the remaining non-recombinant subgenotypes were always greater than 4% (Table 3).

**Table 3:** Mean percentage of nucleotide divergences over the entire genome of HBV between different non-recombinant subgenotypes in the novel classification.

	C10	C12	C11	C6	tentativeC14 (C×4)	C1	C8	Quasi-subgenotype C2	C9	C7	C3	C13	C15	C16
C10		0.5	0.6	0.5	0.4	0.4	0.5	0.4	0.5	0.5	0.5	0.5	0.6	0.6
C12	7.1		0.4	0.3	0.3	0.4	0.4	0.3	0.3	0.4	0.3	0.4	0.4	0.4
C11	6.9	4.8		0.3	0.4	0.4	0.4	0.4	0.4	0.4	0.4	0.4	0.4	0.4
C6	6.8	4.9	4.2		0.4	0.4	0.4	0.3	0.3	0.4	0.3	0.3	0.4	0.4
tentativeC14 (C×4)	5.2	6.0	6.0	6.0		0.3	0.4	0.3	0.3	0.3	0.3	0.4	0.4	0.4
C1	5.5	6.3	6.1	6.2	4.8		0.4	0.3	0.4	0.4	0.4	0.4	0.5	0.4
C8	6.6	5.3	5.3	5.2	5.8	5.7		0.3	0.4	0.4	0.4	0.4	0.5	0.5
Quasi-subgenotype C2	5.4	5.2	5.1	5.0	4.4	4.5	4.6		0.3	0.3	0.3	0.3	0.4	0.4
C9	6.0	4.9	4.8	4.8	5.1	5.4	4.6	4.3		0.4	0.4	0.4	0.4	0.4
C7	5.9	5.2	5.2	5.3	5.2	5.1	4.6	4.1	4.2		0.4	0.4	0.4	0.5
C3	6.8	5.1	4.8	4.7	5.7	5.8	5.0	4.8	4.6	5.1		0.4	0.4	0.4
C13	7.4	5.9	4.9	5.0	6.9	6.6	6.1	5.9	5.7	6.2	5.9		0.4	0.4
C15	7.3	5.2	4.7	5.0	6.1	6.2	5.4	5.3	4.9	5.5	5.0	5.3		0.5
C16	6.8	4.6	4.6	4.7	5.8	5.7	5.3	5.2	5.0	5.3	5.1	5.5	5.2	

## Discussion

The accurate classification of genotype and subgenotype of HBV is important in that different viral genotypes and subgenotypes have shown differences in the course of disease, responses to anti-viral treatment regimens, and in clinical outcomes [4], [6], [35]–[39]. For example, subgenotype B1 was related to fulminant HBV infections in Japan. However, subgenotype B2 has been reported to be associated with HCC or HCC recurrence in young patients in East Asia [40], [41]. In particular, both subgenotypes C1 and C2 have been reported to be associated with the risk of hepatocellular carcinoma (HCC). However, only C2 has been associated with an increased risk of HCC [42].

It is still controversial whether recombinants should be reported separately or designated as novel subgenotypes [8]. Although inclusion of the recombinant sequences into phylogenetic analysis did change the topology of the tree, it played a limited role in subgenotyping the non-recombinant sequences. In addition, there haven’t been generally accepted rules for reporting HBV recombinants by far. Therefore, the designation of subgenotypes C4, C5, CD1 and CD2 remained unchanged, although all of them have been proven as inter-genotype recombinants. The C/D recombinants have been reported to be specifically restricted to the Qinghai-Tibet Plateau in western China [43]. However, one CD2 virus has also been isolated from Belgium [44], and a few CD1 strains have been isolated from Mongolia (Figure 1) [45].

Our results showed that most of the subgenotypes were properly designated, such as C1, C3, C6 to C13, and C15 to C16. They were monophylies and sequence divergences between them were always greater than 4%. Therefore, no change has been made to these subgenotypes in the new classification.

However, a few misclassifications have been identified and corrected. For example, subgenotype information extracted from GenBank for a few sequences isolated from China was wrong and has been identified to be B/C recombinants in our previous report [28]. C11 proposed by Utsumi and colleagues has been classified into C12 [20]. Two previously designated C6 sequences have been renamed as C12 and C7 respectively.

In particular, subgenotype C2 has been associated with an increased risk of HCC [42]. However, subgenotype C2 was not a monophyly [9]. Furthermore, the classification of the sequences falling between C2 and C1 was problematic and some of them haven’t been designated a subgenotype (Figure 2). To correct the misclassifications in subgenotype C2, we named several subgenotypes, from C×1 to C×9, temporarily. Although some of them (e.g. C×3) were monophylies with high bootstrap support, sequence divergences between C2 and C×1 to C×9 were mostly smaller than 4%. Therefore, designating them as separate subgenotypes was not suitable.

Alternatively, we proposed that quasi-subgenotype C2 should be used. The term “quasi-subgenotype” has been used to correct the misclassifications in the subgenotyping of HBV of genotypes A and B [8], [46], [47]. The novel quasi-subgenotype C2 was composed of sequences of Asian origin and included the old C2, C×1 to C×3, and C×6 to C×9. It also included previously classified C14. However, both advantages and disadvantages of the designation of quasi-subgenotype C2 were distinct. On one hand, introducing the quasi-subgenotype C2 was the simplest, but a feasible way to provide a robust and consistent classification for genotype C HBV, instead of introducing more subgenotypes which would make the HBV subgenotyping classification more complex and inconsistent. On the other hand, the quasi-subgenotype C2 was still not a monophyly, which is contradictory to the current criteria used for HBV subgenotyping.

In addition, C×4 showed more than 4% divergence with the remaining subgenotypes. It was a monophyly with a bootstrap value of 100%. Therefore, we proposed that C×4 should be classified as a novel subgenotype and has been named as the new C14 for continuous numbering.

Based on the above corrections, we propose a novel classification for subgenotyping the genotype C HBV. In the new classification, original C1, C3 to C10, C11 proposed by Mulyanto and colleagues [19], C12 to C13, C15 to C16, CD1 and CD2 remained unchanged. C11 proposed by Utsumi and colleagues [20] are classified as C12. The original C2 has been named as quasi-subgenotype C2 and it included several undefined sequences, as well as previously defined C14. In addition, C×4 has been identified to be a novel subgenotype and has been named as the new C14 for continuous numbering. This new classification system is well supported by the sequence divergence data (Table 3).

Based on the present large-scale analysis, we propose that it should be extremely cautious to propose novel HBV subgenotypes. Apart from phylogenetic analysis and sequence divergence analysis, geographical information and even ethnic information might be used to guide HBV subgenotyping, since distributions of different HBV genotypes and subgenotypes show distinct geographical and certain ethnic characteristics [48]. In addition, most previous analyses with a few selected representative strains often showed high bootstrap support for subgenotype C2 and its monophyletic nature. However, when all genotype C sequences were analyzed together, neither the high bootstrap support for subgenotype C2 nor its monophyletic nature was really guaranteed. Therefore, we suggest that if possible, the designation of a novel subgenotype should be based on a comparison of all available relevant sequences in public databases rather than only a few representative strains.

To sum up, we studied the influence of inclusion of recombinant sequences in the HBV subgenotyping and highlighted the importance and urgency to introduce a novel nomenclature system to report HBV recombinants. In addition, we identified and corrected several misclassifications in the subgenotyping of genotype C HBV. Based on these corrections, a novel, but more robust and consistent classification for the subgenotyping of genotype C HBV has been proposed, in which a novel quasi-subgenotype C2 and a novel subgenotype (new C14) were introduced.

## Supporting Information

Figure S1 **Phylogenetic tree constructed using all genotype C HBV sequences.**
(PNG)

Figure S2 **Phylogenetic tree constructed using all non-recombinant genotype C HBV sequences.**
(PNG)
