# Supplementary figures and images for: Subgenotyping of Genotype C Hepatitis B Virus: Correcting Misclassifications and Identifying a Novel Subgenotype
Source: PLoS One. 2012 Oct 15;7(10):e47271. doi: 10.1371/journal.pone.0047271 (PMC3471840; doi:10.1371/journal.pone.0047271)

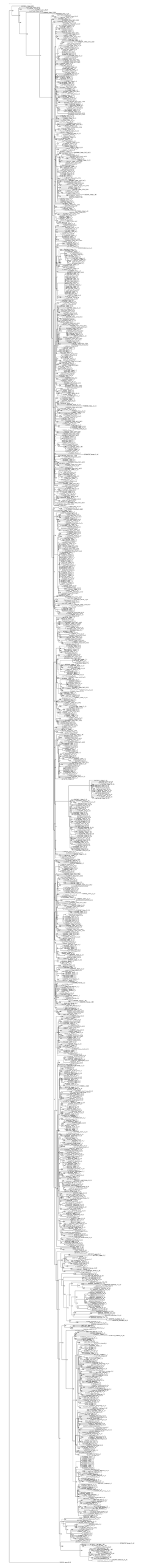

Supplement: Figure S1 — Phylogenetic tree constructed using all genotype C HBV sequences. (PNG) [file pone.0047271.s001.png]

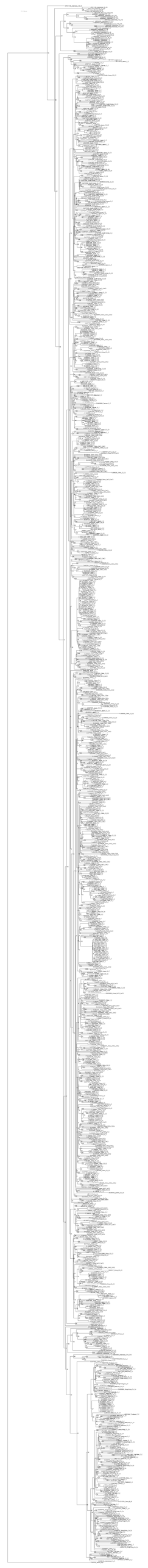

Supplement: Figure S2 — Phylogenetic tree constructed using all non-recombinant genotype C HBV sequences. (PNG) [file pone.0047271.s002.png]
